# Relationship Between Plasma Olanzapine and N-Desmethyl-Olanzapine Concentration and Metabolic Parameters in Patients With Schizophrenia

**DOI:** 10.3389/fpsyt.2022.930457

**Published:** 2022-06-21

**Authors:** Huimei An, Hongzhen Fan, Yajun Yun, Song Chen, Siyuan Qi, Botao Ma, Jing Shi, Zhiren Wang, Fude Yang

**Affiliations:** HuiLongGuan Clinical Medical School, Beijing HuiLongGuan Hospital, Peking University, Beijing, China

**Keywords:** therapeutic drug monitoring, olanzapine, N-desmethyl-olanzapine, metabolic abnormalities, schizophrenia

## Abstract

**Objectives:**

The aim of the present study was to investigate a potential relationship between metabolic parameters and steady-state plasma concentrations of olanzapine (OLA) and its metabolite, 4-N'-desmethyl-olanzapine (DMO) in patients with schizophrenia taking therapeutic doses.

**Methods:**

A total of 352 inpatients, diagnosed with schizophrenia according to the DSM-V criteria and treated with OLA, were investigated. The plasma concentrations of OLA and DMO were measured by high-performance liquid chromatography-mass spectrometry (HPLC-MS/MS). Fasting blood samples were measured for insulin, glucose, total cholesterol (TC), triglycerides (TG), high-density lipoprotein cholesterol (HDL-c), low-density lipoprotein cholesterol (LDL-c), C-reactive protein (CRP) and homocysteine, and differences in these parameters were investigated in relation to plasma concentrations of OLA and DMO.

**Results:**

Lower plasma DMO concentrations were associated with higher glucose and TG levels and homeostasis model assessment of insulin resistance (HOMA-IR), while higher plasma OLA concentrations were associated with higher CRP and homocysteine levels in the OLA-treated patients with schizophrenia.

**Conclusion:**

These results demonstrate that OLA and its metabolite DMO may have different effects on OLA-induced metabolic abnormalities. DMO might have a counteracting effects on glucose-insulin homeostasis and lipid metabolic abnormalities, which suggests that regular measure of various metabolic parameters and drug monitoring on both OLA and DMO are recommended in OLA-treated patients with schizophrenia.

## Introduction

Olanzapine (OLA) is one of the most prescribed atypical antipsychotics for the treatment of schizophrenia. It has a lower incidence of extrapyramidal adverse effects and better therapeutic efficacy than other antipsychotics (1), but is associated with a higher propensity for metabolic abnormalities (2). Therefore, the superiority in clinical efficacy of OLA should be weighed against the metabolic adverse effects when comparing OLA with other antipsychotics (3).

OLA has a high affinity for various receptors (4), and is extensively metabolized after oral administration. It is primarily metabolized by glucuronyltransferase to OLA 10-N-glucuronide, by flavin-containing monooxygenase system to OLA N-oxide, by the major pathway of CYP1A2 to 4-N'-desmethyl-olanzapine (DMO), and by the minor pathway of CYP2D6 to 2-hydroxymethyl OLA (5). Even though OLA 10-N-glucuronide is the most abundant metabolite, DMO is reported to be more strongly associated with the clearance of OLA (6).

Numerous studies have investigated the associations between serum or plasma concentrations of OLA and metabolic abnormalities, including weight gain, impaired glucose metabolism, diabetes mellitus, and dyslipidemia (7–11). A previous review reported a dose-response relationship between OLA serum concentrations and metabolic outcomes (9). Furthermore, a recent study reported the high prevalence of prediabetes and metabolic abnormalities among the schizophrenia patients treated with OLA, putting them at high risk for later cardiovascular disease and type 2 diabetes (12).

In addition to the parent drug OLA, some studies have focused on the relationship between the OLA metabolite DMO and metabolic abnormalities. A recent animal study further reported that DMO can induce a remarkable loss of body weight and fat mass in obese mice and improve insulin resistance and energy expenditure, suggesting that DMO might directly impact the metabolic, endocrinal, and inflammatory effects of OLA (13). Lu et al. reported that in humans, a DMO plasma concentration >5.63 ng/mL is a negative predictor of metabolic syndrome, while C_OLA_/C_DMO_ ratio between 3 and 6 might maximize the therapeutic efficacy and minimize the metabolic adverse effects in OLA-treated schizophrenia patients (14, 15). Although a number of studies have investigated the relationship between DMO plasma concentrations and body weight, waist circumference, and some metabolic parameters, their results are inconsistent (7, 16), and the exact effects of DMO on various metabolic parameters are still unknown. Thus, the aim of this study was to investigate the potential relationships between metabolic parameters and steady-state plasma concentrations of OLA and DMO in schizophrenic patients on therapeutic doses.

## Materials and Methods

### Participants

A total of 325 inpatients who fulfilled the DSM-V diagnostic criteria of schizophrenia were recruited in this retrospective study. All were stable schizophrenic inpatients receiving a fixed dose of OLA (5–20 mg/day) orally for at least 6 months. The daily doses were determined by the physician who was treating the patients. The following information: demographic data, prescribed dose, time of last dose adjustment, time of last drug intake, date of blood sampling, psychiatric diagnosis, and concomitant medication were obtained from the medical record system. Patients treated with sustained-release OLA, carbamazepine and fluvoxamine and other antipsychotics were excluded from the study. In addition, patients with substance addiction, known diabetes mellitus or other physical disease that could influence metabolic parameters were excluded. The study was approved by the Review Board of Beijing Hui-Long-Guan Hospital. Written informed consent for therapeutic drug monitoring (TDM) of OLA was not required as it was a part of clinical routine of blood test.

### Blood Sampling

Blood samples were drawn in the morning generally between 7 and 8 a.m. under fasting conditions, ~12 h after the last dose of OLA. The blood samples were subjected to either biochemical analysis or immediately centrifuged for 10 min at 3,000 g at 4°C. The plasma samples were collected and stored at −20°C until analysis for determination of OLA and DMO, which was completed within 5 days.

### Determination of the Plasma Concentrations of OLA and DMO

The steady-state plasma concentrations of OLA and DMO were measured by the high-performance liquid chromatography-tandem mass spectrometry (HPLC-MS/MS) method, which was detailed in our previous study (17). Briefly, plasma samples of 100 μl were extracted with 300 μl acetonitrile (containing 15 ng/ml d_8_-olanzapine) by agitation for 30 s and centrifuged for 10 min, and then 150 μl of supernatant was injected into the HPLC-MS/MS system. The calibration curves of OLA and DMO was linear in the concentration range of 3.2–160 and 1.6–80 ng/ml, respectively. The detection limits of OLA and DMO were 0.4 and 0.3 ng/ml, respectively. The intra and inter-day precision and relative errors were < 15%.

### Determination of Metabolic Parameters

The serum levels of insulin, glucose, total cholesterol (TC), triglycerides (TG), high-density lipoprotein cholesterol (HDL-c), low-density lipoprotein cholesterol (LDL-c), C-reactive protein (CRP) and homocysteine were measured by commercially available kits (Leadman Biotechnology Co Ltd, Beijing, China) using an automatic biochemistry analyzer AU2700 (Olympus, Japan). The homeostasis model assessment of insulin resistance (HOMA-IR), was calculated using the equation HOMA-IR = fasting insulin (mU/L)^*^fasting glucose (mmol/L)/22.5.

### Statistical Analyses

Statistical analyses were performed using the SPSS 20.0. Kolmogorov-Smirnov test to analyze the normality. Descriptive statistics were presented as the mean ± standard deviation (SD) for continuous variables and the rate for discrete variables. The dose-corrected plasma concentrations of OLA (C_OLA_/D), DMO (C_DMO_/D), and the C_DMO_/C_OLA_ ratio were calculated. Spearman's rank order correlation analysis was used to assess the correlation between variables. Modified Bonferroni's method was used to correct possible errors during multiple tests (18). Further, multiple regression analysis (enter method) was performed to assess the association of each metabolic parameter and C_OLA_ and C_DMO_ after controlling for confounding factors, including age, gender, weight, and dose and duration of OLA. The significance level was set to *p* < 0.05.

## Results

### Demographic and Metabolic Characteristics

A total of 352 inpatients with schizophrenia under TDM for OLA were included in this study. Table 1 shows the demographic and metabolic characteristics of the study participants. The daily OLA dose was positively correlated with C_OLA_ (*r* = 0.567, *p* < 0.001) and C_DMO_ (*r* = 0.569, *p* < 0.001), respectively. C_DMO_ was significantly correlated with C_OLA_ (*r* = 0.606, *p* < 0.001)_._ Age was negatively correlated with the daily OLA dose (*r* = −0.355, *p* < 0.001), C_OLA_ (*r* = −0.176, *p* < 0.001) and C_DMO_ (*r* = −0.258, *p* < 0.001), respectively. Weight was positively correlated with the daily OLA dose (*r* = 0.106, *p* = 0.048), but negatively correlated with C_OLA_ (*r* = −0.114, *p* = 0.034) and C_DMO_ (*r* = −0.212, *p* < 0.001). There were no sex differences in the daily OLA dose and C_DMO_ (both *p* > 0.05), but female patients showed significantly higher C_OLA_ than male patients (57.8 ± 27.2 ng/ml for male and 69.4 ± 30.5 ng/ml for female, *p* < 0.001).

**Table 1:** Demographic and metabolic characteristics of study subjects.

**Parameters**	***n*** **= 352**
Gender (M/F)	241/111
Age (Years)	56.6 ± 14.2
Weight (kg)	67.8 ± 12.5
BMI (kg/m^2^)	23.8 ± 4.4
OLA dose (mg)	14.3 ± 5.0
C_OLA_ (ng/ml)	61.5 ± 28.8
C_DMO_ (ng/ml)	7.1 ± 3.8
C_OLA_/D (ng/ml/mg)	4.5 ± 1.9
C_DMO_/D (ng/ml/mg)	0.5 ± 0.2
The C_DMO_/C_OLA_ ratio	0.1 ± 0.1
Insulin (pmol/L)	57.8 ± 39.9
Glucose (mmol/L)	5.1 ± 1.3
TC (mmol/L)	4.1 ± 0.8
TG (mmol/L)	1.5 ± 0.7
HDL-c (mmol/L)	1.0 ± 0.2
LDL-c (mmol/L)	2.4 ± 0.6
CRP (mg/L)	2.8 ± 5.18
Homocysteine (umol/L)	27.6 ± 17.2
HOMA-IR	1.9 ± 1.5
Comedication (*n*%)	
None	64 (18.2%)
Antidepressant drugs	70 (19.9%)
Mood stabilizing drugs	63 (18.0%)
Antiparkinsonian drugs	91 (25.9%)
Anxiolytic drugs and drugs for sleep disorders	161 (46.0%)
Others	17 (4.8%)

*C_OLA_, concentrations of olanzapine; C_DMO_, concentrations of N-desmethyl olanzapine; D, dose; TG, triglycerides; TC, total cholesterol; HDL-c, high-density lipoprotein cholesterol; LDL-c, low-density lipoprotein cholesterol; CRP, C-reactive protein; HOMA-IR, homeostasis model assessment of insulin resistance*.

### Relationship Between OLA, DMO and Glucose, Insulin

Simple correlation analysis revealed that C_OLA_ was positively correlated with insulin levels, glucose levels, and HOMA-IR. After Bonferroni's correction, only the correlation of C_OLA_ with HOMA-IR remained statistically significant (*p* < 0.05).

Simple correlation analysis revealed that C_DMO_/D and C_DMO_/C_OLA_ ratios were negatively correlated with insulin levels, glucose levels, and HOMA-IR. After Bonferroni's correction, these negative correlations remained statistically significant (all *p* < 0.05) (Table 2).

**Table 2:** Correlations of C_OLA_, C_DMO_, C_OLA_/D, C_DMO_/D and the C_DMO_/C_OLA_ ratio with metabolic parameters.

	**C_**OLA**_**	**C_**DMO**_**	**C_**OLZ**_/D**	**C_**DMO**_/D**	**The C_**DMO**_/C_**OLZ**_ ratio**
**Insulin**					
*r*	0.218	−0.108	0.158	−0.273	−0.366
*P*	0.013	0.220	0.075	0.002^*^	0.001^*^
**Glucose**					
*r*	0.108	−0.102	0.084	−0.173	−0.214
*p*	0.043	0.058	0.120	0.001^*^	0.001^*^
**HOMA-IR**					
*r*	0.260	−0.120	0.173	−0.318	−0.404
*p*	0.003^*^	0.175	0.051	0.001^*^	0.001^*^
**TG**					
*r*	0.096	−0.164	0.022	−0.285	−0.284
*p*	0.075	0.002^*^	0.678	0.001^*^	0.001^*^
**TC**					
*r*	0.109	0.125	0.115	0.128	0.038
*p*	0.042	0.019	0.033	0.017	0.483
**HDL-c**					
*r*	−0.018	0.117	−0.017	0.156	0.174
*p*	0.733	0.030	0.758	0.004^*^	0.001^*^
**LDL-c**					
*r*	0.138	0.110	0.173	0.127	−0.018
*p*	0.010	0.040	0.001^*^	0.019	0.736
**Homocysteine**					
*r*	0.025	−0.138	0.185	−0.039	−0.208
*p*	0.688	0.027	0.003^*^	0.539	0.001^*^
**CRP**					
*r*	0.130	−0.013	0.265	0.032	−0.173
*p*	0.036	0.834	0.001^*^	0.610	0.005^*^

* *p remained statistically significant after Bonferroni's correction*.

Further, multiple regression analysis revealed that lower C_DMO_ was associated with higher glucose levels (β = −0.212, *t* = −2.710, *p* = 0.007) and higher HOMA-IR (β = −0.298, *t* = −2.071, *p* = 0.042) after controlling for age, gender, weight, and dose and duration of OLA.

### Relationship Between OLA, DMO, and Lipids

Simple correlation analysis revealed that C_OLA_ was positively correlated with TC and LDL-c levels; C_DMO_ positively correlated with TC, HDL-c, and LDL-c levels but negatively with TG levels. After Bonferroni's correction, only the negative correlation of C_DMO_ with TG levels remained statistically significant (*p* < 0.05).

Simple correlation analysis revealed that C_OLA_/D was positively correlated with TC and LDL-c levels; C_DMO_/D positively correlated with TC, HDL-c, and LDL-c levels but negatively with TG levels; C_DMO_/C_OLA_ ratio positively correlated with HDL-c levels but negatively with TG levels (Table 2). After Bonferroni's correction, the positive correlation of C_OLA_/D with LDL-c levels and negative correlations of C_DMO_/D and C_DMO_/C_OLA_ ratio with TG and HDL-c levels remained statistically significant (all *p* < 0.05).

Further, multiple regression analysis revealed that lower C_DMO_ was associated with higher TG levels (β = −0.232, *t* = −3.168, *p* = 0.002) after controlling for age, gender, weight, and dose and duration of OLA.

### Relationship Between OLA, DMO and Homocysteine, CRP

Simple correlation analysis revealed that C_OLA_ was positively correlated with CRP levels; C_DMO_ negatively correlated with homocysteine levels. However, neither of the two correlations passed Bonferroni's correction.

Simple correlation analysis revealed that C_OLA_/D was positively correlated with homocysteine and CRP levels, but the C_DMO_/C_OLA_ ratio was negatively correlated with both. After Bonferroni's correction, these correlations remained statistically significant (all *p* < 0.05) (Table 2).

Further, multiple regression analysis revealed that higher C_OLA_ was associated with higher homocysteine (β = 0.245, *t* = 2.556, *p* = 0.011) and CRP levels (β = 0.254, *t* = 2.535, *p* = 0.012) after controlling for age, gender, weight, and dose and duration of OLA.

## Discussion

Metabolic abnormalities are associated with OLA treatment in patients with schizophrenia. The present study found that higher plasma C_OLA_ was associated with higher homocysteine and CRP levels, while lower plasma C_DMO_ was associated with higher glucose and TG levels and HOMA-IR. These results suggest that OLA and its metabolite DMO may have different effects on OLA-induced metabolic abnormalities. DMO might have a counteracting effects on glucose-insulin homeostasis and lipid metabolic abnormalities.

The first finding of the present study is that lower plasma C_DMO_ was associated with higher glucose and TG levels and HOMA-IR in OLA-treated patients with schizophrenia. The findings are in accordance with those of previous studies by Melkersson (7, 16). Similarly, another study observed negative correlations of C_DMO_ with glucose levels, and C_DMO_/D with insulin levels (14). Additionally, a recent animal study found that DMO improves insulin resistance and energy expenditure in mice with high-fat-diet–induced obesity (13). Although it failed to pass Bonferroni's correction, this study found that C_OLA_ was positively correlated with glucose, insulin levels and HOMA-IR. Patients receiving OLA exhibited significantly higher insulin and glucose levels than those receiving conventional antipsychotics, indicating that OLA has a direct stimulating role on insulin secretion from pancreatic beta cells, or may simultaneously act secondary to insulin resistance (19, 20). Thus, we speculated that OLA and DMO could have different effects on glucose-insulin homeostasis, the concentration-dependent metabolic abnormalities of OLA might be complicated by the counteracting effects of DMO (9). Lu et al. previously reported that C_OLA_ ≥ 22.77 ng/mL is a predictor of moderate clinical efficacy, C_DMO_ > 5.63 ng/mL is associated with reduced risk of metabolic syndrome, and C_OLA_/C_DMO_ ratio between 3 and 6 may maximize the therapeutic efficacy and minimize the metabolic adverse effects in OLA-treated patients with schizophrenia (15, 21). Therefore, these results demonstrated that drug monitoring of both OLA and DMO is a valuable method to improve the therapeutic efficacy and decrease the metabolic adverse effects.

The second finding of the present study is that higher plasma C_OLA_ was associated with higher CRP levels. Similarly, a recent study found a trend toward a positive correlation between serum C_OLA_/D and CRP levels in a naturalistic setting (22). C-reactive protein (CRP) is a systemic inflammatory marker associated with obesity, insulin resistance, and cardiovascular disease (23). Previous studies have reported that CRP levels are higher in patients treated with OLA (24–26); however, it is still uncertain whether this is directly associated with OLA treatment or indirectly associated with OLA-induced side effects. Interestingly, a recent meta-analysis reported that CRP levels were increased in patients with schizophrenia but is not altered by antipsychotic medication, notwithstanding whether these were typical or atypical antipsychotics (27). Therefore, although CRP levels were found to be correlated with C_OLA_ in this study, the correlation should be interpreted with more caution.

The third finding of the present study is that higher plasma C_OLA_ was associated with higher homocysteine levels in the OLA-treated patients with schizophrenia. Few studies have explored the association between homocysteine levels and plasma C_OLA_ or C_DMO._ The only study to do so reported a positive correlation between C_OLA_ and homocysteine levels and a marginally positive correlation between C_DMO_ and homocysteine levels (14). Homocysteine is a risk factor for cardiovascular disease and is associated with a variety of neurological and psychiatric diseases (28–30). Numerous studies have further reported elevated homocysteine levels in first-episode (31–33) and chronic schizophrenia patients (29, 34, 35). Elevated homocysteine levels are considered to be a pathophysiological symptom of schizophrenia. Several studies have previously investigated the effects of antipsychotics on homocysteine levels in schizophrenia, but with inconsistent results. Some studies reported that homocysteine levels were decreased after antipsychotic treatment in schizophrenia (31), others found that the homocysteine levels did not change (36). Furthermore, Dicker-Brown et al.'s study demonstrated that glucose and insulin might influence homocysteine metabolism, possibly by affecting the activity of cystathionine-beta-synthase and methylenetetrahydrofolate reductase (37). Therefore, we speculated that the possible explanations for the association between the plasma C_OLA_ and homocysteine levels might be related to the impaired glucose–insulin homeostasis in the OLA-treated patients with schizophrenia.

There are several limitations to the present study. First, this was a retrospective study, several potential confounders, such as diet, exercise, co-medication and disease severity were not considered. Therefore, these correlations should be interpreted with more caution. Second, in the present study, only OLA and its metabolite DMO were analyzed, the possible role of other metabolites (38) on glucose-insulin homeostasis and lipid metabolism therefore could not be evaluated. Third, although a number of significant correlations were found, with plasma C_OLA_ generally being associated with a worsening of metabolic parameters and plasma C_DMO_ the opposite, the strength of these associations was not particularly strong. It is still not enough to accurately clarify the effect of DMO on metabolic parameters in patients with schizophrenia treated with OLA based on available clinical data. Further studies in animal are needed to better understand the metabolic effects of DMO.

## Conclusion

In conclusion, the present study found that higher plasma C_OLA_ was associated with higher CRP and homocysteine levels, and lower plasma C_DMO_ was associated with higher glucose and TG levels and HOMA-IR in the OLA-treated patients with schizophrenia. These findings demonstrate that DMO might counteract the effects on OLA-induced metabolic abnormalities, which suggests that maintaining C_DMO_/C_OLA_ ratio in a suitable range may minimize the metabolic side effects in OLA-treated patients with schizophrenia. Therefore, regular measure of various metabolic parameters and drug monitoring on both OLA and DMO are recommended for patients receiving OLA treatment.

## Data Availability Statement

The original contributions presented in the study are included in the article/supplementary material, further inquiries can be directed to the corresponding authors.

## Ethics Statement

The studies involving human participants were reviewed and approved by the Review Board of Beijing Hui-Long-Guan Hospital. Written informed consent for participation was not required for this study in accordance with the national legislation and the institutional requirements.

## Author Contributions

FY and HA were responsible for the study design, manuscript preparation, and providing the funding for the study. YY, SC, BM, and ZW were responsible for recruiting the patients and collecting clinical data. SQ and JS were responsible for drug assays. HF was responsible for statistical analysis. All authors have contributed to and have approved the final manuscript.

## Funding

This study was supported by grants from Beijing Municipal Science & Technology Commission No. Z191100006619020. The supporters had no role in the design, analysis, interpretation, or publication of this study.

## Conflict of Interest

The authors declare that the research was conducted in the absence of any commercial or financial relationships that could be construed as a potential conflict of interest.

## Publisher's Note

All claims expressed in this article are solely those of the authors and do not necessarily represent those of their affiliated organizations, or those of the publisher, the editors and the reviewers. Any product that may be evaluated in this article, or claim that may be made by its manufacturer, is not guaranteed or endorsed by the publisher.
